# A spectrophotometric method for the determination of tryptophan following oxidation by the addition of sodium hypochlorite pentahydrate

**DOI:** 10.1371/journal.pone.0279547

**Published:** 2023-01-26

**Authors:** Sho Hosokawa, Tatsuya Morinishi, Kazuaki Ohara, Kentaro Yamaguchi, Satoshi Tada, Yasunori Tokuhara

**Affiliations:** 1 Department of Medical Technology, Ehime Prefectural University of Health Sciences, Ehime, Japan; 2 Department of Medical Technology, Kagawa Prefectural University of Health Sciences, Kagawa, Japan; 3 Faculty of Pharmaceutical Sciences at Kagawa Campus, Tokushima Bunri University, Kagawa, Japan; Tribhuvan University, NEPAL

## Abstract

Tryptophan (Trp) is an essential amino acid that functions in various biological processes and human daily health. As the significant functions of Trp become more apparent, its measurement is becoming increasingly important in various situations. Herein, we improved the Trp color reaction based on the Hopkins-Cole reaction and established a simple colorimetric method for Trp determination using several different reagents, including sodium hypochlorite pentahydrate and monosodium glutamate. The detection method can be performed using safe materials, rather than conventional toxic substances, and induces a crimson color change with an absorption peak at 525 nm, enabling the quantification of Trp by simple spectrophotometry in just 10 min. This assay exhibited a linear detection range from 10 to 100 mg/L (R^2^ = 0.9996). The average recoveries in the spiked cerebrospinal fluid ranged from 90.5% to 104.3%, with a relative standard deviation of 0.27% (n = 3, 29.40 mg/L Trp) to 1.19% (n = 3, 72.90 mg/L Trp). This novel spectrophotometric method may enable many researchers and laboratory technicians to detect Trp in various sample solutions without expensive analytical instruments or complicated operations.

## Introduction

Tryptophan (Trp) is one of the essential amino acids that must be obtained through the diet. Beyond its role in protein synthesis, Trp and its metabolites serve functions in daily health such as sleep, circadian rhythm, and cognition [1–3]. Furthermore, changes in the body concentration and metabolism of Trp are associated with cancer, infection, stress, and depression [4–7]. As the significant functions of Trp become more apparent, its measurement technique is becoming increasingly important in various situations [8].

Although multiple sophisticated techniques are available for the determination of Trp and their metabolites, such as high-performance liquid chromatography (HPLC) with mass spectrometry and fluorescence detection, these methods require expensive analytical equipment, complicated operation, and a lot of time for preparation and maintenance [9–12]. Conversely, spectrophotometry involves easy handling and inexpensive equipment that requires almost no maintenance; therefore, it remains a popular method and has been installed in many automated biochemical analyzers. Moreover, most of the colorimetric detection methods involve oxidation of the Trp condensation products with various aldehydes and have long been used for the determination of Trp, such as the Hopkins-Cole reaction [13, 14]. However, the previously established methods may still be time-consuming, require inconvenient sample treatments, rely on unstable colored reactions, or use a hazardous chemical such as concentrated sulfuric acid [15–18]. Thus, more rapid, simple, safe, and accurate methods for Trp detection should be developed.

Sodium hypochlorite pentahydrate (NaOCl·5H_2_O) is a novel oxidant, often provided in a pure solid form (finely ground) [19]. In previous experiments, we have developed spectrophotometric detection methods of amino acid metabolites using NaOCl·5H_2_O and clarified its usefulness as an oxidizing agent in terms of high stability, quick reaction, and ease of adjustment [20, 21]. Therefore, we attempted to improve the Trp color reaction based on the Hopkins-Cole reaction using NaOCl·5H_2_O as the oxidizing agent.

In the present study, we evaluated the color reaction of Trp solution using NaOCl·5H_2_O and determined the optimized condition for establishing the practical Trp detection method. Using NaOCl·5H_2_O and monosodium glutamate (MSG), a stable crimson color change of Trp solution was observed at 525 nm by spectrophotometry. The optimized Trp colorimetric method was employed for interference and recovery tests of pool specimens, which detected varying Trp concentrations in cerebrospinal fluid (CSF) with little interference. The results may contribute to the development of an accurate, rapid, and stable Trp detection method.

## Materials and methods

### Reagents and apparatus

Asparagine, glycine, alanine, arginine, aspartic acid, cysteine, glutamine, glutamic acid, histidine, isoleucine, leucine, lysine, methionine, phenylalanine, proline, serine, threonine, tyrosine, valine, Trp, 10% hydrochloric acid (HCl), sodium hydroxide (NaOH), ascorbic acid (AA), sodium chloride (NaCl), D (+)-glucose, lysozyme, and glyoxylic acid monohydrate were purchased from Wako Pure Chemical Industries, Ltd. (Osaka, Japan). MSG monohydrate and NaOCl·5H_2_O were purchased from Nacalai Tesque (Kyoto, Japan). Pooled Human Cerebrospinal Fluid (CSF) was purchased from Lee Biosolutions, Inc. (USA, Missouri).

A block incubator, WSC-2620 PowerBLOCK (ATTO Corporation, Tokyo, Japan), was used to set and maintain the temperature (10, 20, 30, 40, 50, 60, and 70°C) of sample solutions.

A double-beam spectrophotometer U-2900 (Hitachi High-Technology Co., Ltd., Tokyo, Japan) with microcells and a 10-mm path length was used to measure the absorption spectra of sample solutions.

### Reagents and standards

The solutions were prepared from high-purity analytical reagents and distilled water. Trp was prepared at 1000 mg/L by dissolution in distilled water, and the solution was diluted to 10, 20, 30, 40, 50, 75, 100, 200, 300, 400, and 500 mg/L with distilled water. The standard amino acids were prepared at 2 g/L by dissolution in distilled water and then diluted to 100 mg/L. Several standard amino acids with low solubility (aspartic acid, glutamic acid, and tyrosine) were stirred and dissolved to a saturated state using a magnetic stirring bar, which was rotated at 200 rpm for one hour at room temperature, and then 100 mg/L solutions were separately prepared. A mixture of 19 standard amino acids was prepared by mixing equal amounts (2 g/L) of each (asparagine, glycine, alanine, arginine, cysteine, glutamine, histidine, isoleucine, leucine, lysine, methionine, phenylalanine, proline, serine, threonine, valine) and saturated amino acid solutions (aspartic acid, glutamic acid, and tyrosine), except Trp. MSG was prepared at 500 g/L by dissolution in distilled water, and the solution was diluted to 50, 100, 200, 300, and 400 g/L with distilled water. NaOCl·5H_2_O solution (20 wt%) was prepared by mixing water with NaOCl·5H_2_O at a ratio of 1:5 (w/w), and the solution was diluted to 1, 3, 5, and 10 wt% with distilled water. NaCl was prepared at 300 g/L by dissolution in water. The MSG-NaCl mixture was obtained by mixing 400 g/L of MSG and 300 g/L of NaCl at a ratio of 10:9.

### Procedure spectrophotometric

A total of 600 μL of 10 to 500 mg/L of Trp, 100 mg/L of 19 other standard amino acids, or 100 mg/L of 19 other standard amino acids mixture was added to a test tube, followed by 100 to 500 g/L of MSG (100 μL) or 200 g/L of glyoxylic acid monohydrate (100 μL), and then 10% HCl (10 μL) and 1 to 20 wt% NaOCl·5H_2_O (10 μL) were added. After these well-mixed solutions were incubated at a preset temperature (10–70°C) for 10 min, the absorption spectra (400 to 700 or 800 nm) and absorbance values at 525 nm were measured. Similarly, Trp was combined with several concentrations of AA (25, 50, 100, and 200 mg/L), glucose (25, 50, 100, and 200 mg/L), glyoxylic acid monohydrate (25, 50, 100, and 200 mg/L), or lysozyme (100, 300, 500, and 1000 mg/L) and evaluated to examine the effects of coexisting materials. A total of 600 μL of Trp solutions (50 mg/L) containing each interference substance was added to a tube, followed by 400 g/L of MSG (100 μL), 10% HCl (10 μL), and 5 wt% NaOCl·5H_2_O (10 μL). These well-mixed solutions were incubated at 10°C for 10 min, and then the absorbance values at 525 nm were measured. Samples were prepared for measuring calibration curves and recovery rates by diluting solutions 5 times with the MSG-NaCl mixture to mitigate the effects of an interfering substance. Prior to sample dilution, one volume of Trp standard solution was added to nine volumes of CSF. A total of 600 μL of sample solution was added to a test tube, followed by 10% HCl (10 μL) and 5 wt% NaOCl·5H_2_O (10 μL), and then mixed well. The sample solutions were incubated at 10°C for 10 min, and then the absorbance values at 525 nm were measured. All measurements were performed with a 1-nm bandwidth at a scan speed of 100 nm/min.

## Results and discussion

### Applicability of NaOCl·5H_2_O for tryptophan colorimetric reaction

**Fig 1:**
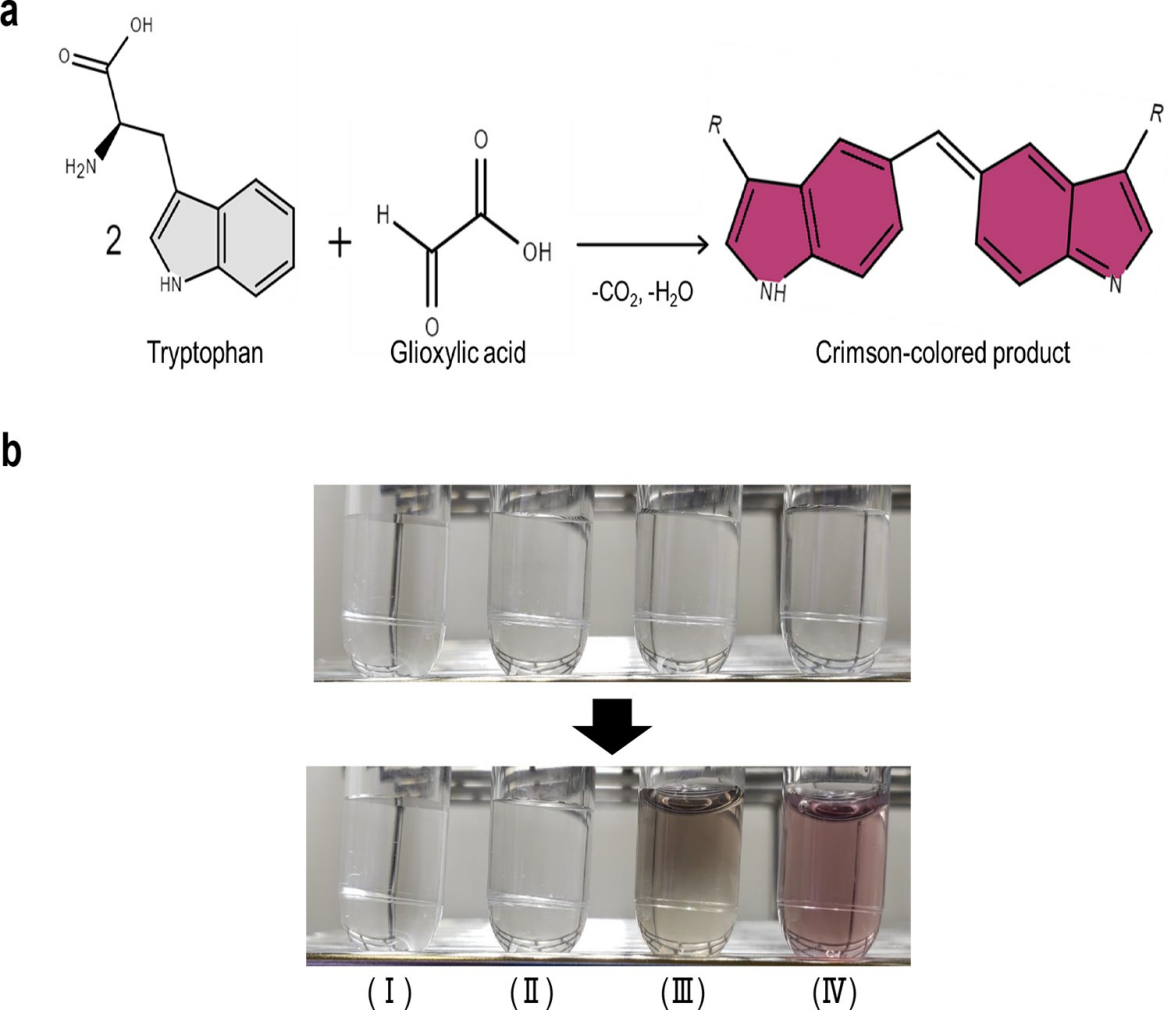
Color reaction of Trp by oxidative condensation with aldehydes. (a) Schematic illustration of Trp coloring reaction with aldehyde based on the Hopkins-Cole reaction. (b) Color reaction of Trp with different reaction solutions: (I) reagent blank (0 mg/L of Trp with 200 g/L of glyoxylic acid monohydrate, 10% HCl, and 3% NaOCl·5H_2_O), (II) 100 mg/L of Trp with 200 g/L of glyoxylic acid monohydrate and 10% HCl, (III) 100 mg/L of Trp with 10% HCl and 3% NaOCl·5H_2_O, and (IV) 100 mg/L of Trp with 200 g/L of glyoxylic acid monohydrate, 10% HCl, and 3% NaOCl·5H_2_O.

In the Hopkins-Cole reaction [13, 14], crimson pigment is produced due to the oxidation reaction of Trp and glyoxylic acid, which is an aldehyde compound, by sulfuric acid (Fig 1A). Considering this, we oxidized Trp and glyoxylic acid using a novel reagent as an alternative oxidant, namely NaOCl·5H_2_O, and a crimson color change was observed after the oxidation (Fig 1B) [22]. However, Trp solution without glyoxylic acid also showed a slight color change after the oxidation with NaOCl·5H_2_O (Fig 1B). A previous study reported that free amino acids were decarboxylated and deaminated by the addition of NaOCl, and aldehydes were generated [22]. Therefore, as shown in Fig 1B, the oxidation and color reaction of Trp with aldehydes, which are decomposition products of the reaction between Trp and NaOCl, may occur without glyoxylic acid. In addition, the Trp solution with a mixture of 19 standard amino acids, instead of glyoxylic acid, exhibited a brilliant crimson color change following the addition of NaOCl·5H_2_O (S1A Fig), and highly concentrated Trp solutions also showed the same color development without glyoxylic acid (S1B Fig). Although conventional Trp coloring methods, such as the Hopkins-Cole reaction [13, 14], directly add the aldehyde reagent to the Trp solution, they have low reproducibility because of the instability of aldehydes and the side reactions caused by excess aldehyde reagent [23, 24]. To overcome these problems, Kibrick adopted a method using methyl alcohol as an aldehyde source and observed a stable color reaction by gradually oxidizing methyl alcohol to formaldehyde and suppressing the generation of unintended byproducts in the reaction solution [23]. Nevertheless, other problems remain unsolved such as requiring excessive time and hazardous chemicals. In this study, we generated a stable Trp coloring reaction in only 10 min using NaOCl·5H_2_O. This method may enable safer, shorter, and more stable color development than previous methods.

### Evaluation of additive reagents

**Fig 2:**
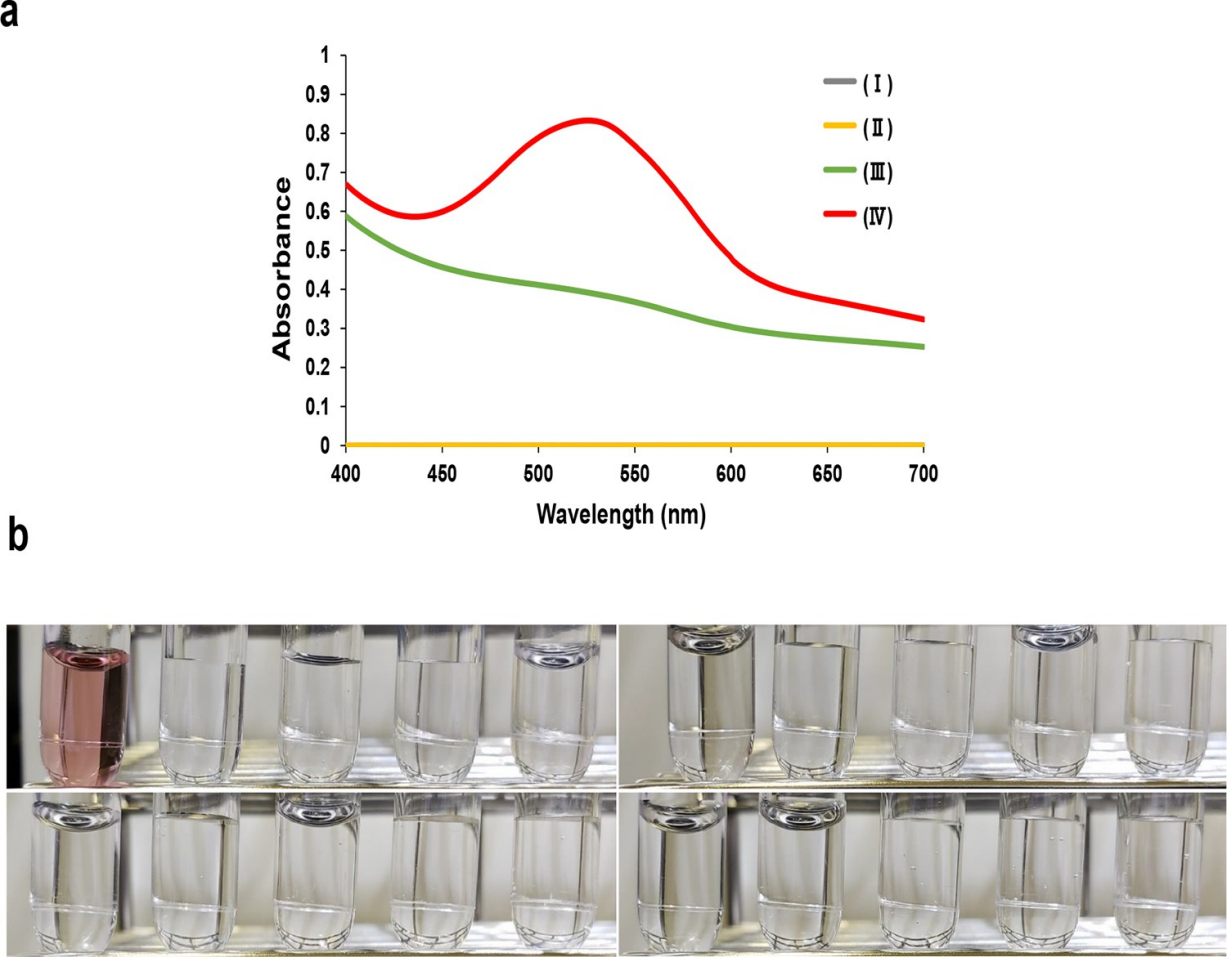
Color changes and absorption spectra of Trp in water with MSG. (a) Absorption spectra of Trp color reaction with different reaction solutions: (I) reagent blank (0 mg/L of Trp with 200 g/L of MSG, 10% HCl and 3% NaOCl·5H_2_O), (II) 100 mg/L of Trp with 200 g/L of MSG and 10% HCl, (III) 100 mg/L of Trp added with10% HCl and 3% NaOCl·5H_2_O, and (IV) 100 mg/L of Trp with 200 g/L of MSG, 10% HCl and 3% NaOCl·5H_2_O. (b) Color reaction for 100 mg/L of 20 standard amino acids with 200 g/L of MSG, 10% HCl, and 3% NaOCl·5H_2_O. From upper left to right: Trp, Ser, Gly, Ala, Leu, Arg, Met, His, Thr, and Ile. From lower left to right: Pro, Phe, Lys, Val, Cys, Gln, Glu, Asn, Asp, and Tyr.

A previous study reported that the aldehyde was formed from glutamic acid because of oxidation caused by NaOCl·5H_2_O [25]. Therefore, we focused on MSG, which is a sodium salt of glutamic acid, as an alternative aldehyde source. We then evaluated the color changes and absorption spectrum (400–700 nm) of Trp solution with MSG following the addition of NaOCl·5H_2_O under acidic conditions. As shown in Fig 2A, the solution showed an absorbance peak at 520–540 nm, even with low Trp concentrations. Additionally, to examine the specificity of the Trp colorimetric reaction, 19 other amino acids were studied. As shown in Fig 2B, none of the 19 amino acids exhibited a color reaction. Therefore, the oxidation of MSG by NaOCl·5H_2_O specifically contributes to the coloring reaction of Trp, indicating that MSG is a useful aldehyde source.

**Fig 3:**
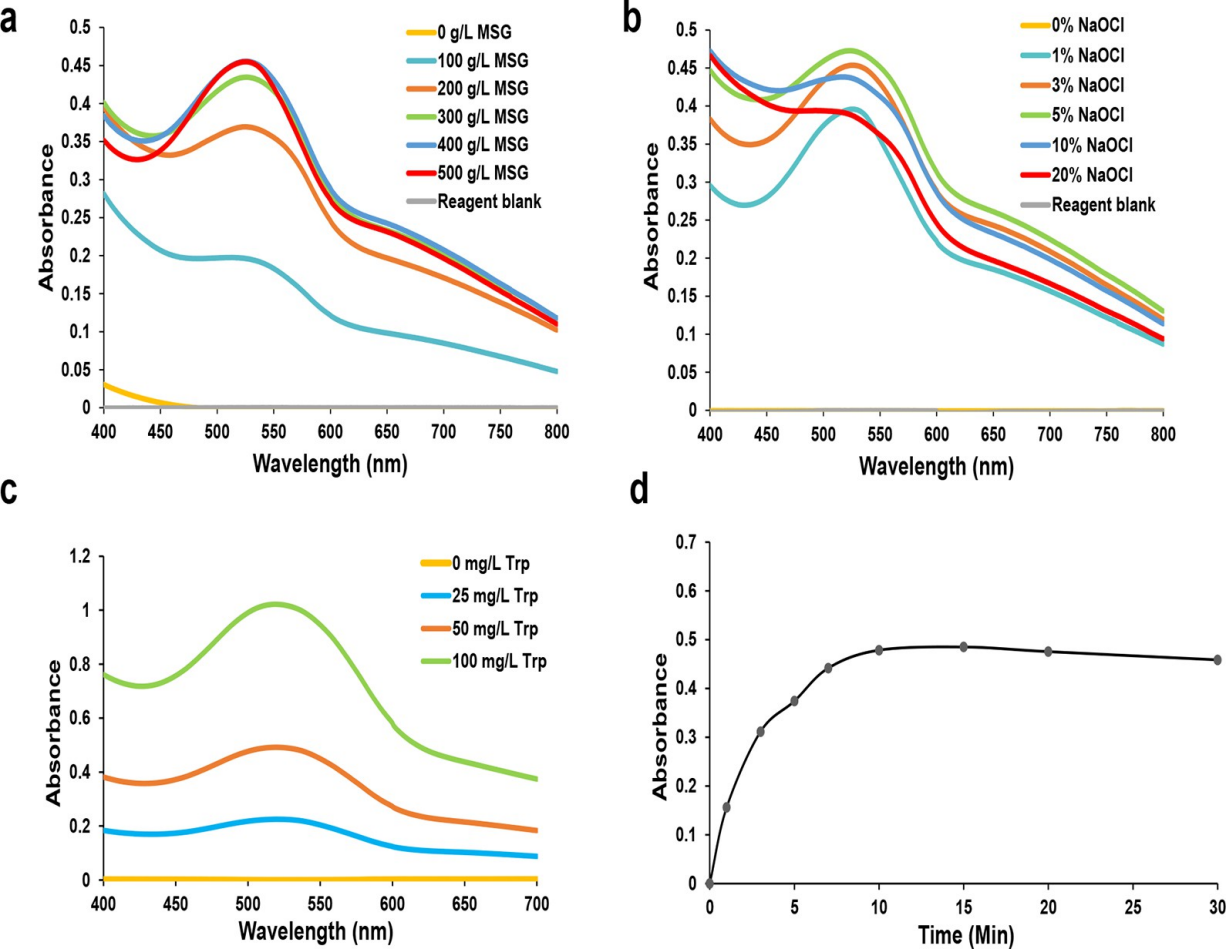
Optical absorption spectra versus concentration of additive reagent. (a) Absorption spectra for 50 mg/L of Trp and different concentrations of MSG (100, 200, 300, 400, and 500 g/L) with the addition of 10% HCl and 3% NaOCl·5H_2_O. Reagent blank (0 mg/L of Trp with 400 g/L of MSG, 10% HCl, and 3% NaOCl·5H_2_O). (b) Absorption spectra for 50 mg/L of Trp with 400g/L of MSG, 10% HCl, and several concentrations of NaOCl·5H_2_O (1, 3, 5, 10, and 20%). Reagent blank (0 mg/L of Trp with 400 g/L of MSG, 10% HCl, and 3% NaOCl·5H_2_O). (c) Absorption spectra for various concentrations of Trp (0, 25, 50, and 100 mg/L) after adding 400 g/L of MSG, 10% HCl, and 5% NaOCl·5H_2_O. (d) Change in the absorption intensity (525 nm) of Trp solution (50 mg/L) over time (1, 3, 5, 7, 10, 15, 20, and 30 min), after adding 400 g/L of MSG, 10% HCl, and 5% NaOCl·5H_2_O.

To develop a more stable and sensitive color reaction, different reagent concentrations were examined. First, we added 100–500 g/L of MSG to Trp solutions (50 mg/L) and measured changes in the absorption spectrum (400–800 nm) following the addition of NaOCl·5H_2_O under acidic conditions. The absorbance peak at 525 nm increased in intensity with increasing MSG concentration (Fig 3A), reaching saturation at 400 g/L of MSG, and the excess never inhibited its color reaction.

One of the advantages of NaOCl·5H_2_O is the ease of adjustment due to the solid form (finely ground powder). To assess this, NaOCl solutions between 1 and 20% (w/w) concentration were prepared and added to Trp solution containing MSG. The absorption spectra (400–800 nm) exhibited a characteristic peak at 525 nm that originally increased with increasing NaOCl concentration (Fig 3B). However, the peak intensity decreased when the oxidant concentration exceeded 10%, and the spectra became broader. Since excessive oxidation may be available to trigger other reactions [26], the rate of oxidation also needs to be controlled by the proper adjustment of NaOCl·5H_2_O.

Next, we examined the relation between Trp concentrations and absorption spectra. The solutions with 25, 50, and 100 mg/L of Trp were incubated with MSG, HCl, and NaOCl·5H_2_O for 10 min. The absorption spectra with a peak around 525 nm decreased in a dose-dependent manner (Fig 3C). In addition, we also examined the color stability of the reaction. From Fig 3D, the absorbance intensity at 525 nm for 50 mg/L of Trp solution increased for 10 min after the reaction began and remained stable for at least 20 min more. These results suggest that this spectrophotometric method may have the potential to quantify the concentrations of Trp.

### Evaluation of pH and temperature

**Fig 4:**
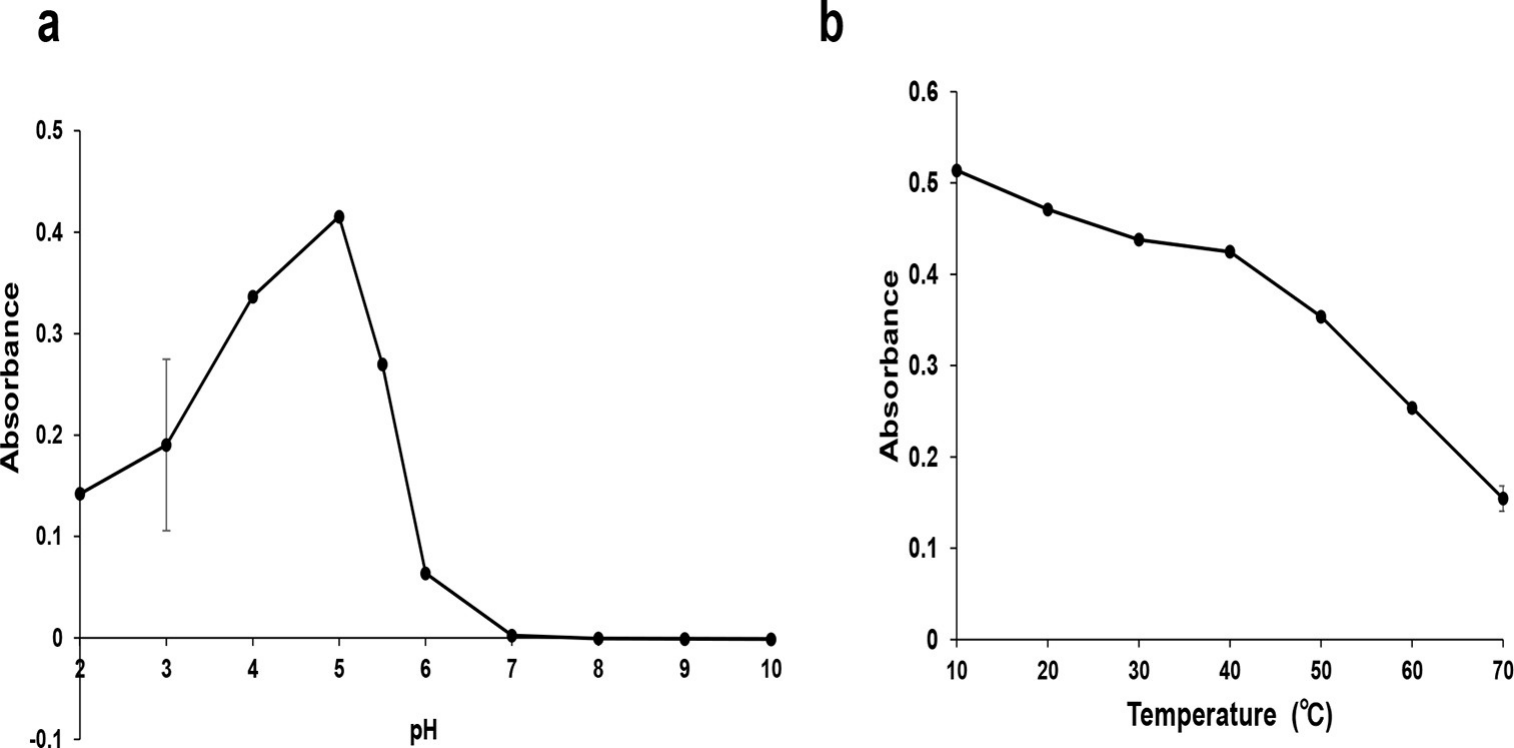
Effects of temperature and pH on absorbance values of Trp. Change in the absorption intensity (525 nm) of Trp solution (50 mg/L) (a) at different pH and (b) different temperatures, after the addition of 400 g/L of MSG, 10% HCl, and 5% NaOCl·5H_2_O. Results are the mean ± S.D. of three experiments.

Another experiment was conducted to examine the effects of pH on the reaction. The assay was conducted using mixed solutions containing Trp and HCl or NaOH, with an adjusted pH ranging from 10 to 2. As shown in Fig 4A, the Trp solution did not develop any color under neutral to alkaline conditions. The absorbance was highest at pH 4–5 but then decreased under more acidic conditions (pH < 4). These results demonstrated that the optimal pH for the Trp color reaction was weakly acidic.

Next, the effects of temperature were investigated by performing the reaction at 10 to 70°C. The maximum color development was obtained at 10°C, and the color change was diminished by an increase in temperature (Fig 4B). At a high temperature, the reaction products obtained by the condensation of Trp with aldehyde compounds may not be homogeneous and these byproducts can increase with the temperature [24, 27]. Overall, the temperature and pH influence the reaction. Considering the instability and harm of operating under high temperatures and strong acidic conditions, the color reaction is preferably conducted under weakly acidic and low-temperature conditions.

### Interference effects

**Fig 5:**
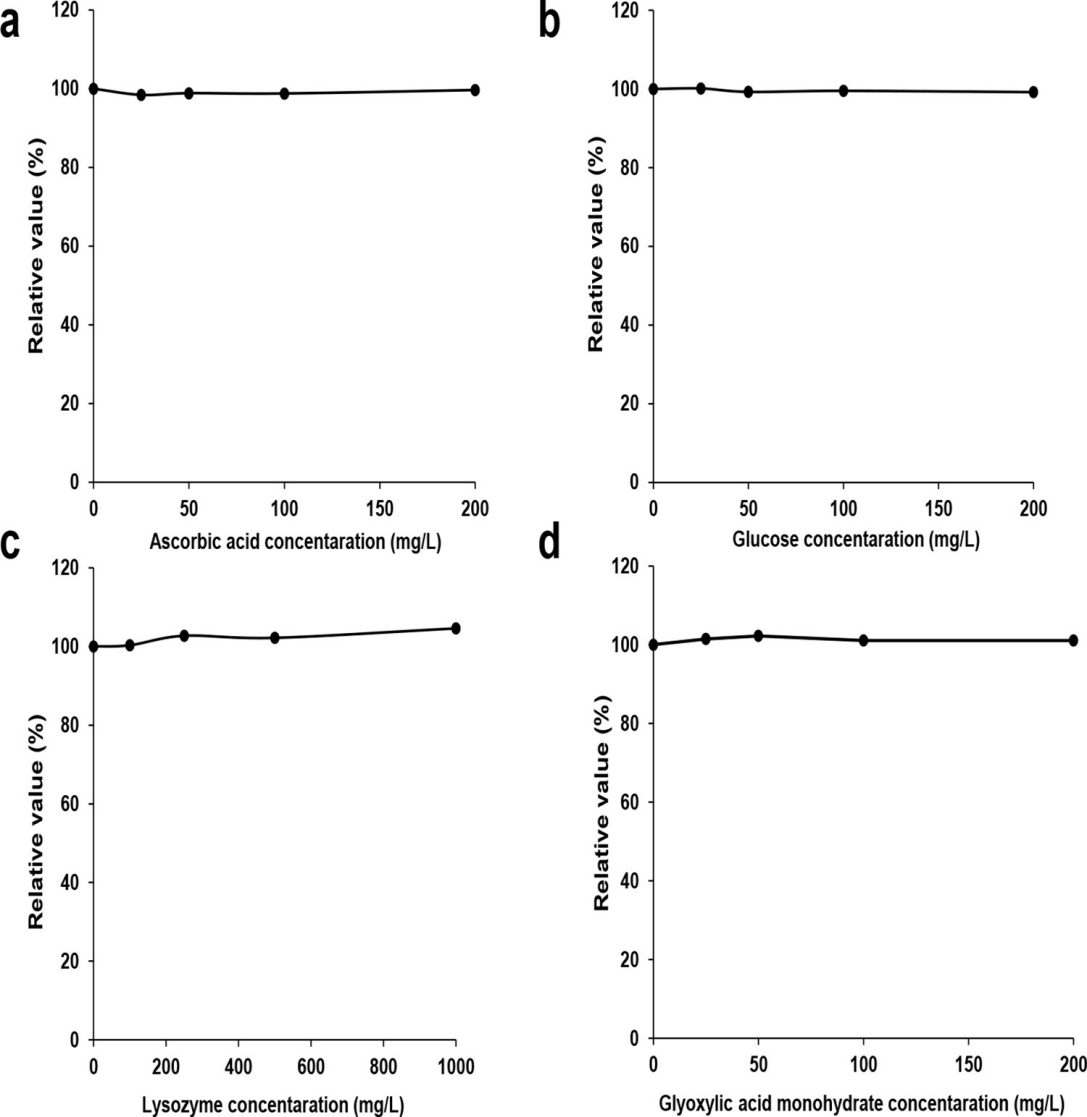
Effects of interference substances on absorbance values of Trp. Change of the absorption intensity (525 nm) of Trp solution (50 mg/L) containing several concentrations of (a) AA, (b) glucose, (c) lysozyme, and (d) glyoxylic acid, after the addition of 400 g/L of MSG, 10% HCl, and 5% NaOCl·5H_2_O.

In biological samples, food, and beverages, Trp coexists with various substances such as other amino acids, aldehyde compounds, and antioxidants. To confirm whether our colorimetric reaction is stable under various practical conditions, we investigated the influence and potential interference of coexisting materials. Since our method is dependent on oxidation, we investigated whether the antioxidant action of AA affected the Trp absorption peak at 525 nm. Although the rise time of the peak was delayed, interference action was not observed in 10 min with up to 200 mg/L of AA, (S2 Fig, Fig 5A). Similarly, up to 200 mg/L of glucose did not show interference action (Fig 5B). Moreover, the influence of proteins containing Trp on the color reaction was examined. Lysozyme, found in numerous foods and various types of biological samples, is a well-known protein that is high in Trp. As shown in Fig 5C, the absorbance peak was constant, without depending on the concentration of lysozyme up to 1000 mg/L. This result suggests that free Trp contributes to the coloring reaction and Trp-constituting protein is not involved. Moreover, glyoxylic acid relating to the Hopkins-Cole reaction is often included in cosmetics, food flavors, and medicine [24], but its presence (up to 200 mg/L) in the Trp solution did not affect the Trp absorption peak (Fig 5D).

### Calibration curve and recovery rates

**Fig 6:**
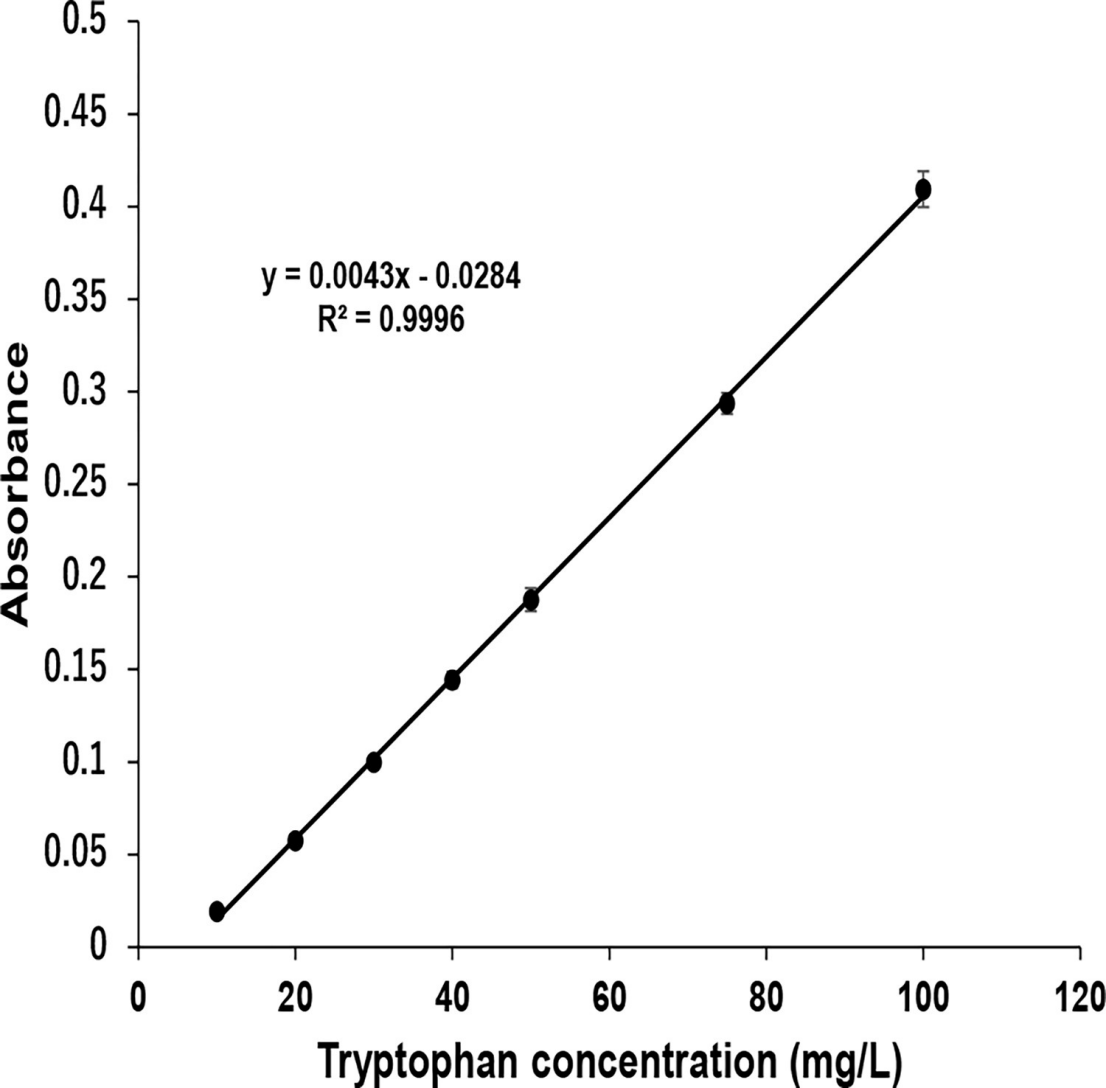
Calibration curve. Correlation between Trp concentration (10 to 100 mg/L) and absorbance (525 nm) under optimized conditions. Results are the mean ± S.D. of three experiments.

Previous studies reported that an altered Trp concentration in CSF and plasma is related to the outcome of diseases and daily health [28–30]. Therefore, to demonstrate the practical application of the optimized colorimetric assay, we prepared a calibration curve, and the accuracy of detecting Trp in pool specimens composed of CSF was verified. As a pretreatment step, all Trp standards in CSF were diluted 5 times with an MSG-NaCl mixture to minimize the impact of pH changes and interferential action by coexisting substances. Under this condition, to prepare the calibration curve, correlations between the Trp concentration and its absorbance were examined following the addition of NaOCl·5H_2_O. From 10 to 100 mg/L, a linear correlation was confirmed (R^2^ = 0.9996) (Fig 6). Based on the data, recovery experiments were performed by spiking different concentrations of Trp standard solution into CSF prior to the pretreatment step. The recovery ratio at two spiked levels was in the range of 90.5% to 104.3% with a relative standard deviation of 0.27% (n = 3, 29.40 mg/L Trp) to 1.19% (n = 3, 72.90 mg/L Trp), which confirmed that the optimized colorimetric assay can detect Trp in spiked pool specimen with little interference (Table 1). Previous reports showed that blood plasma from healthy subjects contained amounts of Trp between 8.8 and 15.2 mg/L [31], and the urinary Trp levels of patients with Hartnup disease, which is an inborn metabolic disorder involving Trp, contained more than 35 mg/L [32]. Although we did not detect concentrations of Trp less than 10 mg/L, absorbance at 525 nm showed a linear trend from 10 to 100 mg/L (Fig 6). To develop a quantitative method for Trp, further examinations to detect Trp solutions containing less than 10 mg/L are required for the verification of several conditions, including the optimal pH and concentrations of MSG and NaOCl·5H_2_O. However, our current results indicate that the spectrophotometric method may be useful for the detection of Trp in biological samples.

**Table 1:** Spectrophotometric determination of Trp in pool specimens (n = 3).

Sample	Spiked concentration(mg/L)	Measured concentration(mg/L)	RSD(%)	Recovery(%)
	0	10.35±0.02	0.11	-
cerebrospinal fluid	30	29.40±0.19	0.27	90.5
	60	72.90±2.12	1.19	104.3

In recent years, the measurement of Trp in food, beverages, and biological samples has become possible using HPLC with mass spectrometry and fluorescence detection, which has the advantages of sensitivity and selectivity [11, 33, 34]. However, these methods require expensive analytical equipment, complicated operation, and excessive time for preparation and maintenance. In contrast, since spectrophotometers are installed in many research institutes and laboratories, Trp can easily be detected using our method under less restricted conditions than HPLC.

To improve the Trp colorimetric reaction in the past, different reagents and conditions have been investigated, and various colorimetric methods have been developed [14, 15, 18]. Recently, Abdulwahed et al. developed an improved Hopkins-Cole reaction for the simple and reliable chromogenic reaction of glycolic acid and tryptophan in the presence of ferric chloride and concentrated sulfuric acid [24]. Huang et al. developed a simple, rapid, and highly selective Trp colorimetric method by improving the oxidation reaction of Trp and formic acid, an aldehyde compound, under acidic conditions [35]. Nevertheless, the use of harmful substances and the effects of interference have not been fully addressed. Conversely, our method does not use toxic materials, such as concentrated sulfuric acid, and all reagents used in this method are inexpensive and readily available. Furthermore, our spectrophotometric method, which is less susceptible to the effects of interfering substances, may enable many researchers and laboratory technicians to detect Trp in a variety of sample solutions without expensive analytical instruments or complicated operations. Therefore, our method can also be automated to measure many samples because it is characterized by simple, stable, and rapid absorption spectroscopy and requires a small sample volume.

## Conclusion

In the present study, we reported a simple, accurate, and rapid method for the colorimetric detection of Trp using NaOCl·5H_2_O and MSG. We examined validation parameters, selectivity, linearity, interference effects, precision, and recovery. Our method can be performed using safe materials, rather than conventional toxic substances, and induces a crimson color change with an absorption peak at 525 nm within 10 min, potentially enabling the quantification of Trp by simple spectrophotometry. These results suggest that our method can help researchers and laboratory technicians to detect Trp in various sample solutions without expensive analytical instruments or complicated operations.

## Supporting information

S1 Fig Color reaction of tryptophan with different reaction solutions. (a) (I) Reagent blank (a mixture of 19 standard amino acids, 10% HCl, and 3% NaOCl·5H_2_O), (II) 100 mg/L of Trp (a mixture of 19 standard amino acids and 10% HCl), (III) 100 mg/L of Trp (10% HCl and 3% NaOCl·5H_2_O), and (IV) 100 mg/L of Trp (a mixture of 19 standard amino acids, 10% HCl, and 3% NaOCl·5H_2_O). (b) (I) Reagent blank (10% HCl and 3% NaOCl·5H_2_O), (II) 400 mg/L of Trp (10% HCl), (III) 400 mg/L of Trp (10% HCl and 3% NaOCl·5H_2_O).
(PDF)

S2 Fig Effect of AA on tryptophan color reaction. Relationship between absorbance intensity at 525 nm and time of incubation in the presence or absence of AA.
(PDF)

S1 Table Absorbance values. Raw absorbance data are available in the excel file.
(XLSX)
