# Supplementary material for: A spectrophotometric method for the determination of tryptophan following oxidation by the addition of sodium hypochlorite pentahydrate
Source: PLoS One. 2023 Jan 26;18(1):e0279547. doi: 10.1371/journal.pone.0279547 (PMC9879471; doi:10.1371/journal.pone.0279547)
Supplement: S1 Fig — (a) (I) Reagent blank (a mixture of 19 standard amino acids, 10% HCl, and 3% NaOCl·5H2O), (II) 100 mg/L of Trp (a mixture of 19 standard amino acids and 10% HCl), (III) 100 mg/L of Trp (10% HCl and 3% NaOCl·5H2O), and (IV) 100 mg/L of Trp (a mixture of 19 standard amino acids, 10% HCl, and 3% NaOCl·5H2O). (b) (I) Reagent blank (10% HCl and 3% NaOCl·5H2O), (II) 400 mg/L of Trp (10% HCl), (III) 400 mg/L of Trp (10% HCl and 3% NaOCl·5H2O). (PDF) [file pone.0279547.s001.pdf]

a

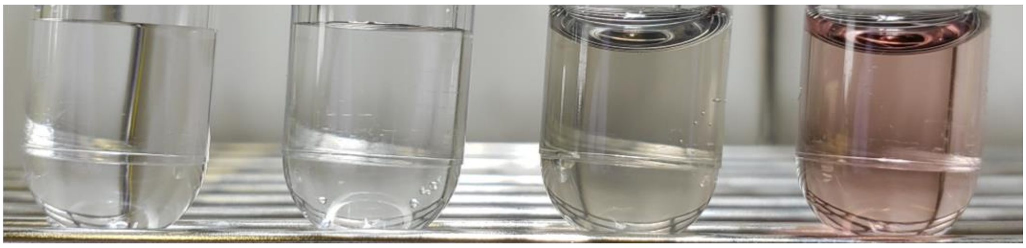

b

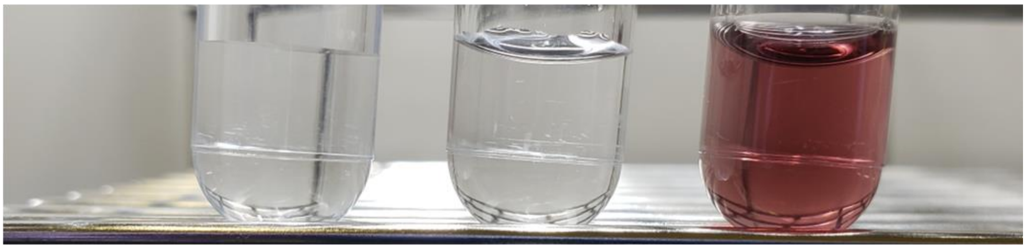

S1 Fig. Color reaction of tryptophan with different reaction solutions.

(a) (I) Reagent blank (a mixture of 19 standard amino acids, 10% HCl, and 3% $NaOCl·5H_2O$), (II) 100 mg/L of Trp (a mixture of 19 standard amino acids and 10% HCl), (III) 100 mg/L of Trp (10% HCl and 3% $NaOCl·5H_2O$), and (IV) 100 mg/L of Trp (a mixture of 19 standard amino acids, 10% HCl, and 3% $NaOCl·5H_2O$). (b) (I) Reagent blank (10% HCl and 3% $NaOCl·5H_2O$), (II) 400 mg/L of Trp (10% HCl), (III) 400 mg/L of Trp (10% HCl and 3% $NaOCl·5H_2O$).
