# Supplementary material for: A spectrophotometric method for the determination of tryptophan following oxidation by the addition of sodium hypochlorite pentahydrate
Source: PLoS One. 2023 Jan 26;18(1):e0279547. doi: 10.1371/journal.pone.0279547 (PMC9879471; doi:10.1371/journal.pone.0279547)
Supplement: S2 Fig — Relationship between absorbance intensity at 525 nm and time of incubation in the presence or absence of AA. (PDF) [file pone.0279547.s002.pdf]

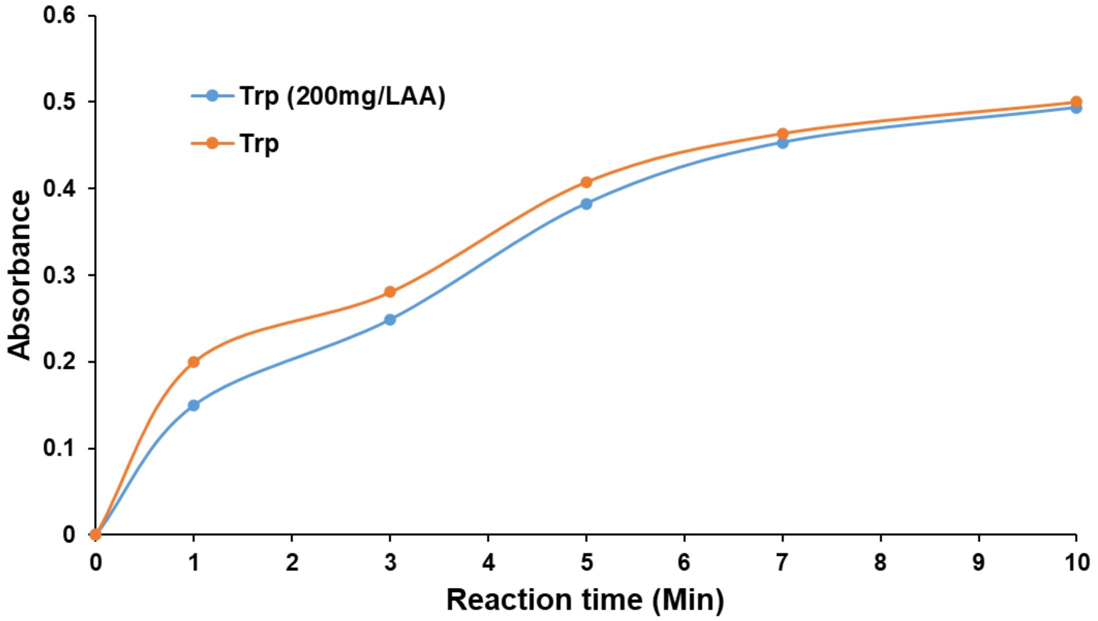

S2 Fig. Effect of AA on tryptophan color reaction.

Relationship between absorbance intensity at 525 nm and time of incubation in the presence or absence of AA.
